# Non-invasive Vagus Nerve Stimulation in Cerebral Stroke: Current Status and Future Perspectives

**DOI:** 10.3389/fnins.2022.820665

**Published:** 2022-02-16

**Authors:** Lijuan Li, Dong Wang, Hongxia Pan, Liyi Huang, Xin Sun, Chengqi He, Quan Wei

**Affiliations:** ^1^Rehabilitation Medicine Center and Institute of Rehabilitation Medicine, West China Hospital, Sichuan University, Chengdu, China; ^2^Key Laboratory of Rehabilitation Medicine in Sichuan Province, Sichuan University, Chengdu, China; ^3^Department of Rehabilitation Medicine, Affiliated Hospital of Chengdu University, Chengdu, China

**Keywords:** non-invasive vagus nerve stimulation, transcutaneous cervical VNS, transcutaneous auricular VNS, rehabilitation, stroke, parameters

## Abstract

Stroke poses a serious threat to human health and burdens both society and the healthcare system. Standard rehabilitative therapies may not be effective in improving functions after stroke, so alternative strategies are needed. The FDA has approved vagus nerve stimulation (VNS) for the treatment of epilepsy, migraines, and depression. Recent studies have demonstrated that VNS can facilitate the benefits of rehabilitation interventions. VNS coupled with upper limb rehabilitation enhances the recovery of upper limb function in patients with chronic stroke. However, its invasive nature limits its clinical application. Researchers have developed a non-invasive method to stimulate the vagus nerve (non-invasive vagus nerve stimulation, nVNS). It has been suggested that nVNS coupled with rehabilitation could be a promising alternative for improving muscle function in chronic stroke patients. In this article, we review the current researches in preclinical and clinical studies as well as the potential applications of nVNS in stroke. We summarize the parameters, advantages, potential mechanisms, and adverse effects of current nVNS applications, as well as the future challenges and directions for nVNS in cerebral stroke treatment. These studies indicate that nVNS has promising efficacy in reducing stroke volume and attenuating neurological deficits in ischemic stroke models. While more basic and clinical research is required to fully understand its mechanisms of efficacy, especially Phase III trials with a large number of patients, these data suggest that nVNS can be applied easily not only as a possible secondary prophylactic treatment in chronic cerebral stroke, but also as a promising adjunctive treatment in acute cerebral stroke in the near future.

## Introduction

It is estimated that there will be approximately 200 million stroke patients in the world by 2050 (Brainin et al., 2020). Despite extensive therapeutic advances in recent years, stroke including ischemic and hemorrhagic (roughly 87 and 13%) (Kuriakose and Xiao, 2020), is still a leading cause of disability and a significant health problem worldwide. Approximately 60% of patients who suffer stroke only partially recover or are unable to recover within 6 months (Lee et al., 2015). Therefore, it is paramount to develop novel complementary treatment approaches that can be easily applied and do not interfere with established protocols including thrombolysis and thrombectomy.

During stroke rehabilitation, developing effective and evidence-based therapies to reduce impairment, improve functional activities, and enhance participation in activities are important goals. Neurostimulation techniques have been used increasingly in clinical and fundamental neuroscience. Vagus nerve stimulation (VNS), a Food and Drug Administration (FDA)-approved addition to medication for the treatment of partial epilepsy, depression, and primary headache disorders, is one potential therapy (Ben-Menachem, 2002; Yuan and Silberstein, 2016; Carreno and Frazer, 2017). It has also recently been recognized that VNS has the potential to enhance the recovery from neurological injuries, including stroke (Khodaparast et al., 2014, 2016; Capone et al., 2017; Dawson et al., 2021). The VNS-REHAB study, which was recently published in the Lancet, supports the use of VNS as a new therapeutic option for limb paralysis caused by an ischemic stroke (Dawson et al., 2021). In clinical practice, two methods of stimulation are used: invasive vagus nerve stimulation (iVNS) and non-invasive vagus nerve stimulation (nVNS) (Mertens et al., 2018; Wang et al., 2021c). nVNS are non-invasive devices that have been developed to stimulate the vagus nerve transcutaneously. By which, unique risks and adverse events associated with implants such as medical care, infection, peritracheal hematoma, damaged vocal cords, and dyspnea are precluded or reduced (Ben-Menachem et al., 2015; Zhao X.-P. et al., 2019; Li et al., 2020b). Furthermore, nVNS delivery systems may be more suitable for emergency patients who have suffered bursts of ischemic stroke. These systems may not require a surgical procedure, thereby improving patient safety.

As nVNS continues to rapidly grow in popularity and application in stroke, the field generally lacks a consensus on optimum initial time, stimulation cites, and stimulation parameters. The question of whether the nVNS can have the same effects in stroke recovery, as well as the underlying mechanisms and future research directions, needs to be addressed further. Therefore, this critical review aims to explore the reported studies on nVNS in stroke to present narrative accounts of its therapeutic potential and mechanisms of action that may facilitate its therapeutic effects. The abbreviations in this review are listed in Table 1.

**TABLE 1:** Abbreviations.

Abbreviations			
Auricular branch of the vagal nerve	ABVN	Middle cerebral artery occlusion	MCAO
Autonomic nervous system	ANS	Myeloperoxidase	MPO
Blood brain barrier	BBB	Non-invasive vagus nerve stimulation	nVNS
Blood oxygen level dependent	BOLD	Non-invasive VNS	nVNS
Brain-derived neurotrophic factor	BDNF	Norepinephrine	NE
Central nervous system	CNS	Nucleus tractus solitarious	NTS
Cholinergic anti-inflammatory pathway	CAP	Percutaneous auricular VNS	paVNS
Dentate gyrus	DG	Peroxisome proliferator-activated receptor γ	PPARγ
Dynamic contrast enhanced MRI	DCE-MRI	Post-stroke insomnia	PSI
Electromyogram	EMG	Spreading depolarization	SD
Endothelial nitric oxide synthase	eNOS	Tight junction protein	TJP
Food and Drug Administration	FDA	Transcutaneous auricular vagus nerve stimulation	taVNS
Fugl-meyer assessment-upper extremity	FMA-UE	Transcutaneous cervical vagus nerve stimulation	tcVNS
Function independent measure	FIM	Transcutaneous vagus nerve stimulation	tVNS
Functional magnetic resonance imaging	fMRI	Traumatic brain injury	TBI
Growth differentiation factor 11	GDF11	Tumor necrosis factor α	TNF-α
Human high mobility group 1	HMGB1	Upper limb fugl-meyer	UFM
Hypothalamic–pituitary–adrenal axis	HPA	Vagus nerve	VN
Interleukin	IL	Vagus nerve stimulation	VNS
Invasive vagus nerve stimulation	iVNS	Vascular endothelial growth factor	VEGF
Ischemia/reperfusion	I/R	Wolf motor function test	WMFT
Matrix metalloproteinase	MMP	α7 nicotinic acetylcholine receptor	α7nAchR

## Vagus Nerve Stimulation

### History and Clinical Application of Vagus Nerve Stimulation

Vagus nerve stimulation has a history dating back to the 19th century when James Corning examined the anti-seizure effect of manual stimulation of the vagal nerve in epileptic patients (Lanska, 2002). There are two methods of stimulation in clinical practice, invasive vagus nerve stimulation (iVNS) and non-invasive vagus nerve stimulation (nVNS). According to an international consensus published recently, there are four currently accepted VNS modalities: cervically implanted VNS (iVNS), transcutaneous cervical VNS (tcVNS), transcutaneous auricular VNS (taVNS), percutaneous auricular VNS (paVNS) (Farmer et al., 2020). In iVNS, a pulse generator is implanted beneath the skin in the upper chest, along with electrodes connected to the left vagal nerve (Goodnick et al., 2001; Pruitt et al., 2016; Dawson et al., 2021). Systems for delivering nVNS utilize the distribution of vagal afferents through the skin, either at the external ear (taVNS) or in the neck (tcVNS) (Straube et al., 2015; Gaul et al., 2016; Genheimer et al., 2017; Burger et al., 2019).

Following decades of trials conducted on animals and humans. iVNS was approved by the FDA for the treatment of medically refractory partial epilepsy in 1997 (Morris et al., 2013) and severe, recurrent unipolar depression and bipolar depression in 2005 (Young et al., 2020). iVNS Therapy also received Conformite Europeenne (CE) marking in Europe for the treatment of epilepsy and treatment-resistant or treatment-intolerant depression (DeGiorgio and Krahl, 2013; Young et al., 2020). Invasive surgeries and their unwanted side effects of iVNS have led to the development of a new, completely non-invasive stimulation way. nVNS has received special attention from basic, clinical, and translational studies due to its comparable benefits to iVNS, ease of use, higher accessibility, and fewer side effects (Ben-Menachem et al., 2015; Frangos et al., 2015; Marin et al., 2018). nVNS entered clinical treatment in 1997, its clinical effectiveness and its physiological action are similar but with greater tolerability and fewer patients reporting side effects (Redgrave J. et al., 2018). tcVNS has also been approved by the FDA to treat migraines (Martelletti et al., 2018) and cluster headaches (Gaul et al., 2016; Marin et al., 2018).

In such a long period of clinical practice, hundreds of thousands of patients have been treated for various neurological disorders, such extensive experience has provided many opportunities to explore new clinical applications for VNS in other neuropsychiatric disorders except epilepsy, migraines, depression. And Among the most intriguing potential directions of VNS is the treatment of stroke. Recent randomized controlled trials have also shown that combined with rehabilitation therapy, iVNS and nVNS may benefit upper limb recovery after stroke (Khodaparast et al., 2014; Capone et al., 2017; Dawson et al., 2021).

### Anatomic Basis for Non-invasive Vagus Nerve Stimulation

The vagus nerve (VN) is a mixed cranial nerve composed of 80% sensory fibers (afferent) and 20% motor fibers (efferent). It is located on both the left and right sides of the body, acting as a two-way channel between the central nervous system and the autonomic nervous system (ANS), transmitting sensory and motor information between the systems. Its afferent fibers transmit visceral and somatic information from the body to the brainstem and thus providing a unique pathway to the brain (Groves and Brown, 2005; Kaniusas et al., 2019; Farmer et al., 2020). While its efferent fibers originate in the dorsal motor nucleus (to supply the heart, lungs, esophagus, and stomach) and in the nucleus ambiguous (to innervate the muscles in pharynx and larynx). Most of afferent fibers of VN terminate in the nucleus tractus solitarius (NTS) in the lower medulla (e.g., for visceral afferents, heart, taste, and aorta), whereas others terminate in the nucleus spinalis of the trigeminal nerve, like some laryngeal and pharyngeal afferents (Trevizol et al., 2015; Yuan and Silberstein, 2016). The right part of the vagus nerve is more closely associated with the cardiac atria and innervates the sinoatrial node that controls heart rate; whereas the left part of the vagus nerve is typically associated with the ventricles of the heart and innervates the atrioventricular node that controls contraction force (Guiraud et al., 2016). The vagus nerve is therefore essential in the maintenance of homeostasis and parasympathetic system function, regulating inflammatory, cardiovascular function, and gastric emptying efferent effects.

According to Erlanger and Gasser, the VN consists of A-, B-, and C-fibers with corresponding conduction velocities (Yuan and Silberstein, 2016). Based on anatomical research, as the VN passes caudally through their ganglia, it divides into four branches: the auricular branch, the meningeal branch, the sympathetic branch (joint with the superior cervical sympathetic ganglion), the pharyngeal branch, and the laryngeal branch (Ruffoli et al., 2011; Yuan and Silberstein, 2016; Kaniusas et al., 2019). The auricular branch of the vagus nerve (ABVN) is the only branch of vagus nerve that reaches the body surface. As the ABVN forms a cutaneous receptive field in the pinna, which is roughly located in the 1–1.5 mm gap between the skin and the auricular cartilage (Bermejo et al., 2017). ABVN can be found in both the cymba and cavum conchae, however, cymba conchae are 100% dominated by ABVN (Peuker and Filler, 2002). The ABVN afferent fiber enters the vagal trunk *via* the jugular ganglion and projects NTS, where the integration of autonomic neurons occurs. The conchae collect afferent information and activate the caudal ventrolateral medulla and dorsal motor nucleus to control central autonomic activity (Butt et al., 2020; Wang et al., 2021b). This is why the conchae have the ability to manage bodily functions. Yakunina et al. (2017) found that stimulation of the auricular canal could activate the vagus nerve pathway to the maximum extent, so this location might be the best anatomical location for transcutaneous vagus nerve stimulation.

During the first half of the twentieth century, researchers began studying the NTS of the vagus nerve, the main afferent transmission from the vagus nerve to the central nervous system, and its projections to the cortex. The areas of the brain that are activated by nVNS depending on the focus have been speculated in various studies. Empirical measures, such as fMRI, EEG, and MEG, are critical to confirm proposed hypotheses (Schulz-Stübner and Kehl, 2011; Colzato et al., 2018; Jongkees et al., 2018). Burger and Verkuil (2018) suggest that nVNS engages limbic areas, such as the hippocampal and amygdala, while Yuan and Silberstein (2016) suggest that stimulation of the vagus nerve influences the distribution of hypocretin and orexin in people with cluster headache, and Jacobs et al. (2015) suggest that nVNS enhances memory by increasing locus coeruleus activity. With fMRI. Kraus et al. (2007) demonstrated that non-invasive vagus nerve stimulation results in prominent changes in cerebral activity with marked deactivation in temporal and limbic regions. fMRI studies have shown that nVNS increases neural activity more than sham stimulation in the left prefrontal cortex, right caudate, mid-cingulate and cerebellum (Badran et al., 2018). It also decreases functional connectivity between the posterior cingulate cortex and the lingual gyrus (Zhao B. et al., 2019), and suppresses processes to generate tinnitus (Yakunina et al., 2018; Yakunina and Nam, 2021).

Stimulation of the vagus nerve may also increase synaptic plasticity in central networks after injury (Meyers et al., 2018; Collins et al., 2021). When the vagus nerve is stimulated electrically, the neuromodulatory effect is immediately triggered. A VNS pulse rapidly activates noradrenergic locus coeruleus and cholinergic nucleus basalis, two key neuromodulators in the brain (Morrison et al., 2021). When these pro-plasticity neuromodulators are simultaneously released with neural activity related to rehabilitation, synaptic plasticity in task-specific circuits is promoted.

In general, VN activity correlates with wellbeing, health, relaxation, and even emotions like empathy, while it is negatively correlated with risk factors such as morbidity, mortality, and stress (Thayer et al., 2010; Zulfiqar et al., 2010; Farmer et al., 2020). VN thus plays a critical role in brain-body interactions. These complex interactions naturally cause interest in artificial stimulation for therapeutic purposes.

## Non-Invasive Vagus Nerve Stimulation in Animal Models of Stroke

In the review of nine animal studies (Ay et al., 2016; Jiang et al., 2016; Ma et al., 2016; Yang et al., 2018; Zhao X.-P. et al., 2019; Li et al., 2020b,a; Lindemann et al., 2020; Zhao et al., 2022; Table 2), most manuscripts have settled on a frequency of 20 or 25 Hz that has been shown to be more biologically active both in implanted functional neuroimaging as well as in taVNS optimization trials (Raedt et al., 2011; Hays et al., 2014; Thompson et al., 2021). The FDA approved areas of 20 to 30 Hz stimulation frequencies because studies had shown that frequencies of 50 Hz and above can cause severe and irreversible damage to the vagus nerve during VNS (Groves and Brown, 2005). Table 2 shows that three studies used the cervical branch of the vagus nerve and six studies used the ABVN as stimulation locations. In rodent models, although the lateral differences are not clear and may differ depending on the parameters used, most of these studies used the left vagus nerve for stimulation.

**TABLE 2:** Stimulation location, parameters, and therapeutic effects for all studies of nVNS in rodent models of stroke.

Authors	Rodent models	Device	Initial time	Parameters	Stimulation side and sites	Stimulation duration	Effects	Results and conclusion
Zhao et al., 2022	Rat, I/R (right ICA)	taVNS, tcvns (Hanshi Electroacupuncture Instrument, Nanjing Hanshi Co. Ltd.)	24 h post-stroke	10 Hz, 1 mA, Pulse width (not described)	Bilateral concha auricularis region or rat tragus	30 min/session, 7 days	Levels of acetylcholine, IL-1β, IL-6, and TNF-α↓; Cx43 phosphorylation↓	Improves motor function
Li et al., 2020a	Rats, MCAO/R (right)	taVNS (Grass Model S48 stimulator, Grass Technologies, Warwick, United States)	30 min post-stroke	20 Hz, 0.5 mA, 0.5 ms, square wave	Left cavum concha	60 min/session, twice daily, 14 days, 28 days	PPAR-γ↓; BDNF, VEGF, P-eNOS↑	Decreases neurological deficit scores, neuronal damage, and infarct volume. Increases microvessel density and endothelial cell proliferation.
Lindemann et al., 2020	Rats, MCAO (left)	tcVNS, iVNS (External transcutaneous stimulator, electrocore Inc.)	30 min post-stroke	iVNS: 25 Hz, 0.5 mA, 0.3 ms tcVNS:25 Hz,1 ms, 5 kHz sine waves.	Left vagus nerve (ivns), left cervical vagus nerve (tcvns)	iVNS: 60 min; tcVNS: 2 min, repeated after 15 min	Spreading depolarizations frequency↓	Improves behavioral tests. Reduces infarct volume. Both iVNS and nVNS reduce the frequency of SDs.
Li et al., 2020b	Rats, MCAO/R (right)	taVNS (Grass Model S48 stimulator, Grass Technologies, Warwick, United States)	30 min post-stroke	20 Hz, 0.5 MA, 0.5 ms, square	Left cavum concha	60 min/session, twice daily,14 days, 28 days	α7nAchR expression↓; Activation of the BDNF/cAMP/PKA/p-CREB pathway	Enhance axonal plasticity through activation of the BDNF/cAMP/PKA/p-CREB pathway
Zhao X.-P. et al., 2019	Mice, MCAO/R (right)	tcVNS (gammacore; Lectrocore, LLC, Basking Ridge, NJ, United States)	1 d before MCAO	25 Hz, 1 ms, 5 kHz sinewaves average voltage of 15 V	Right cervical vagus nerve	60 min	M2 phenotype microglia : Arg-1^+^ cells↑; IL-17A↓; (TUNEL + NeuN+) cells↓	Reduces infarct volume. Improves neurological outcomes. Reduces neurons apoptosis. Promotes microglial M2 polarization.
Yang et al., 2018	Rats, MCAO (right)	taVNS (gammacore; Lectrocore, LLC, Basking Ridge, NJ, United States)	30 min post-stroke	25 Hz, 1 ms, 5 kHz sinewaves average voltage of 15 V	Left cervical vagus nerve	50 min	TJPs: ZO-1↑ BBB transfer rate, serum IgG leakage↓; MMP-2/9 ↓	Reduces infarct volume. Protects Blood-brain barrier.
Ma et al., 2016	Rats, MCAO/R (right)	taVNS (Grass Model S48 stimulator, Grass Technologies, Warwick, United States)	30 min post-stroke	20 Hz, 0.5 mA, 0.5 ms, square	Left cavum concha	60 min/session, twice daily,24 h, 3 days,7 days	upregulate cerebral GDF11 and downregulate splenic GDF11; increase expression of ALK5 in ECs; stimulate proliferation of ecs.	Prompts neuro behavioral recovery Stimulated proliferation of endothelial cells.
Jiang et al., 2016	Rats, MCAO/R (right)	taVNS (Grass Model S48 stimulator, Grass Technologies, Warwick, United States)	30 min post-stroke	20 Hz, 0.5 mA, 0.5 ms, square	Left cavum concha	60 min/session, 2–3 weeks	Microvessel density and endothelial cell proliferation↑; BDNF, eNOS and VEGFs↑	Prompts neuro behavioral recovery and angiogenesis. Reduces infarct volume.
Ay et al., 2016	Rats, MCAO (right)	tcVNS (gammacore; electrocore, LLC).	30 min post-stroke	25 Hz, 1 ms, 5 kHz, 12 V sine waves	Right vagus nerve in the neck	60 min	DecreaseIba-1, CD68, and TNF-α positive cells and increase the number of HMGB1 positive cells.	Reduces infarct volume. Improves neurological score. Inhibits ischemia-induced immune activation.

The tcVNS was initiated at variable times (30 min to 24 h) after cerebral ischemia in rats and mice. The ability of tcVNS to activate the NTS was assessed using c-Fos immunohistochemistry. tcVNS activates the vagus nerve fibers and stimulates its main afferent relay nuclei in the brainstem (NTS) (Ay et al., 2016). The main effects and mechanisms of nVNS illuminated in animal research are summarized below.

### Reducing Infarct Size and Improving Neurological Outcome

Several animal studies have demonstrated that nVNS could reduce the cerebral infarction volume and improve the neurological deficit remarkably in rats with cerebral ischemia (Ay et al., 2016; Zhao X.-P. et al., 2019; Li et al., 2020b; Lindemann et al., 2020; Zhao et al., 2022). In these studies, nVNS provided approximately a 25–50% reduction in infarct size, which was similar to previously reported reductions achieved by iVNS (Ay et al., 2011; Sun et al., 2012). Ay et al. (2016) tested the effect of tcVNS at different initiated time after middle cerebral artery occlusion (MCAO) on tissue and functional outcome by changing the therapeutic window up and down by 1 h each time until a comparable effect size with 30-min stimulation was achieved. They found that the effect of tcVNS on infarct size was consistent when stimulation was initiated up to 4 h after MCAO. Furthermore, the improvement in forelimb function was so long-lasting that it continued even after the stimulation had stopped, consistent with results obtained in aged ischemic stroke rats treated with iVNS and rehabilitative training (Hays et al., 2016).

### Promoting Angiogenesis

After focal cerebral ischemia, the newly formed collateral blood vessels can improve perfusion of the surrounding tissues and promote recovery of nervous system functions. Recent studies have suggested that angiogenesis, almost in parallel to neurogenesis, participates in the recovery of neurological function after ischemic stroke (Song et al., 2019; Alrafiah et al., 2021; Wang et al., 2021a). It was proposed that VNS increased hippocampal progenitor cell proliferation in the adult rat dentate gyrus, so that such progenitor cells contribute to the healing of damaged neurons from ischemic injury (Lu et al., 2017). It would appear that this plasticity is involved in VNS’s efficacy as a treatment for ischemic stroke. In cerebral ischemic rats, taVNS enhanced the expression of angiogenic factors, including BDNF, eNOS, and VEGF, and increased endothelial proliferation, stimulated angiogenesis, and increased microvessel density surrounding the infarct area (Zhang et al., 2017). Another study has shown that taVNS promotes endothelial cells proliferation 7 days after cerebral ischemia, and that taVNS enhances expression of ALK5 in endothelial cells (Ma et al., 2016). The effects of taVNS on post-stroke recovery, as well as up-regulation of cerebral GDF11, and down-regulation of splenic GDF11, indicate brain-spleen communication. Following a stroke, the brain releases ischemic signals, the activated spleen released its GDF11 reserves into the blood circulation, allowing it to deposit in the damaged brain. These results indicate that taVNS may enhance the recovery after stroke by increasing GDF11 concentrations in the vasculature (Ma et al., 2016).

### Regulating Blood Brain Barrier Permeability

The breakdown of the Blood Brain Barrier (BBB) and the subsequent brain edema are two of the key components of neurological dysfunction in stroke. They are associated with poor clinical outcomes during and after ischemic stroke (Cai et al., 2014). A significant association between stroke progression and BBB breakdown has been demonstrated. As early BBB permeability can be reversed with treatment, it would make sense that the VNS could be involved in regulating cerebral edema after stroke (Gaul et al., 2016).

According to a study, the use of taVNS during MCAO significantly reduced the permeability of the BBB after ischemia and reperfusion measured by DCE-MRI 24 h after stroke. taVNS treated rats with ischemic hemispheres demonstrated significantly lower levels of serum IgG leakage as detected by IHC after MRI, consistent with the findings described above (Yang et al., 2018). BBB integrity is maintained primarily by ECs sealed at tight junctions, astrocyte endfeet, pericytes, and extracellular matrix. In reperfusion injury, proteases are involved in the biphasic opening of the BBB. A number of mechanisms have been proposed to account for the degradation of tight junction proteins (TJPs). Matrix metalloproteinases (MMPs) are degrading enzymes that disrupt TJPs, leading to BBB disruption during ischemic stroke. In the ischemic hemisphere, taVNS inhibited BBB breakdown, as evidenced by decreases in TJP cleavage, ZO-1, occludin, and claudin-5 in endothelial cells. Additionally, it protected tight junction proteins in microvessels from disruption and reduced MMP-2/9 expressions in astrocytes around compromised vessels (Yang et al., 2018). In addition, taVNS improved BBB integrity after cerebral cortex microinfarcts as well as in rat models of cortical dysplasia and traumatic brain injury, indicating that it may be useful in the effects on BBB after ischemic stroke.

### Inhibiting Neuroinflammation

Researchers believe that VNS can potentially modulate inflammation *via* a broader vagal neural network (Yuan and Silberstein, 2016). Recent studies suggest that VNS may act as a neuromodulator to activate certain innate, protective pathways in the central nervous system (CNS). VNS may exert its anti-inflammatory properties in a variety of diseases through its afferent fibers (activating the HPA pathway) and efferent fibers (activating the CAP pathway).

The vagus nerve system suppresses the release of proinflammatory cytokines. It was found that VNS reduced plasma levels of TNFα, IL-1β, IL-6, and MPO in colitis rats through the autonomic neural pathway (Sun et al., 2013). There have been animal and clinical studies exploring the efficacy of nVNS in the treatment of inflammation. A study found that taVNS reduced IL-6 and TNF-α release and prevented endotoxemia in mice (Hong et al., 2019). Lerman et al. (2016) found that tcVNS reduced levels of cytokines and chemokines in the blood of healthy people. Meanwhile, Clancy et al. (2014) reported that taVNS decreased sympathetic nerve activity in healthy people.

Through alpha-7 nicotinic acetylcholine receptors (α7nAChRs), central immune activation (e.g., macrophage accumulation and microglial activation) can influence acetylcholine levels and cause anti-inflammatory effects (Kalkman and Feuerbach, 2016). The α7nAChR subunit is required for the CAP to limit cytokine production, according to Wang et al. (2003). The cholinergic anti-inflammatory response is induced by the α7nAChR. Acetylcholine is released when the vagus nerve is stimulated, inhibiting the anti-inflammatory pathway *via* the α7nAChR on activated macrophages and other cytokine-producing cells. Finally, TNF and other pro-inflammatory cytokines that play a role in inflammation are suppressed (Oke and Tracey, 2009). Recent studies have also found that taVNS has anti-inflammatory effects in both the peripheral and central nervous systems, which are mediated through α7nAChRs (Zhao et al., 2012; Corsi-Zuelli et al., 2017). taVNS has also been reported to have neuroprotective effects against ischemic cerebral injuries *via* an anti-inflammatory mechanism (Li et al., 2020b).

Microglia are central nervous system resident macrophages that perform a variety of tasks such as synaptic organization, phagocytosis of apoptotic cells, and neuronal excitability regulation (Sasaki, 2017; Baig et al., 2022). Ischemia triggerS resting microglia to the M1 phenotype causing damage to functioning neural cells including neurons and astrocytes (Hu et al., 2012). Activation of microglia to the M2 phenotype, on the other hand, can stop the inflammatory process by producing anti-inflammatory cytokines like IL-4 and IL-10 (Hu et al., 2012; Liu et al., 2016; Zhao X.-P. et al., 2019). As a result, microglial M2 polarization could be a new target for fighting inflammation after cerebral I/R injury. Zhao X.-P. et al., 2019) demonstrated that tcVNS attenuated cerebral ischemic injury by promoting microglial M2 polarization. Intranasal administration of recombinant IL-17A dampened the tcVNS induced M2 polarization of microglia and its neuroprotective effects, which suggests that the effect of tcVNS might occur through IL-17A signaling inhibition. tcVNS inhibits microglia activation and normalizes altered cytokine levels after MCAO by reducing the number of Iba-1, CD68, and TNF-α positive cells and increasing HMGB1 positive cells (Ay et al., 2016). These findings underline that anti-inflammatory mechanisms play an important role in ischemic neuroprotection by nVNS.

### Facilitating Post-stroke Axonal Plasticity

Axonal plasticity plays an important role in neurofunctional recovery after stroke. The neurofunctional recovery that occurs in the days to weeks following an ischemic stroke appears to be linked to axonal plasticity including axonal regeneration and reorganization (Liu et al., 2015; Bu et al., 2021). taVNS treatment enhanced α7nAchR expression in the ischemic cortex. And ischemic rats treated with taVNS demonstrated improved axonal plasticity (regeneration and reorganization of axons), in accordance with elevated levels of BDNF/cAMP/PKA/p-CREB pathway members. Thus, taVNS could effectively boost axonal plasticity in the brain after I/R injury while improving neurofunctional recovery (Li et al., 2020b).

### Reducing Spreading Depolarizations

Spreading depolarizations (SDs) are sudden and sustained gray matter depolarizations that can occur in a variety of brain states, ranging from healthy brain tissue, such as the migraine aura, to different areas of an ischemic brain, such as the severely energy-depleted infarct core and its surrounding moderately ischemic tissue (Dreier and Reiffurth, 2015). SDs are caused by the failure of the sodium pump in the penumbra aftera n ischemic stroke, and they create cytotoxic edema, disrupt blood flow, and result in infarction of viable tissue, as well as affecting neuronal survival and outcome (Dreier, 2011; Rakers and Petzold, 2017; Dreier et al., 2018; Baig et al., 2022). Furthermore, they are thought to play a role in the development of ionic and vasogenic edema at later stages of ischemia (Dreier et al., 2018; Mestre et al., 2020). As a result, in experimental models and clinical cases of stroke and other acute neurological disorders, SDs are among the most important contributors to infarct generation, cell death, and injury expansion (Lauritzen et al., 2011; Dreier and Reiffurth, 2015). Lindemann et al. (2020) discovered that delivering nVNS or iVNS during permanent MCAO significantly reduced the frequency of SDs in the cortical peri-infarct area compared to sham VNS, without affecting relative blood flow changes, blood pressure, heart rate, or breathing rate. They hypothesize that either nVNS or iVNS could be a safe and effective intervention for reducing the clinical burden of SD waves in stroke.

## Clinical Trials to Assess Safety and Efficacy of Non-Invasive Vagus Nerve Stimulation After Chronic/Subacute Ischemic Stroke

In our review, we found four studies and one case report that investigated the influence of nVNS on upper-limb motor function, sensory function, and sleep disturbance after stroke. Among which, four studies included chronic stroke patients (Capone et al., 2017; Redgrave J.N. et al., 2018; Baig et al., 2019; Zhao B. et al., 2019) except one study included subacute ischemic stroke patients (Wu et al., 2020). Here, we summarized the mainly functional improvement, parameters, side effects and future directions of nVNS in clinical studies on stroke.

In addition, several recently completed and ongoing clinical studies are focused on the safety and effects of nVNS on stroke (Baig et al., 2022). Especially some studies are focused on acute or subacute stroke (NCT03733431; NCT04050501; NCT03292159; Clinicaltrials.gov). Instruments and procedures (MRI, CT perfusion, EMG, or force coupled to a computer monitor) that can help quantify the findings have been utilized in several studies in addition to the generally used scale for outcome evaluation. The findings should help us better understand the effectiveness, adverse effects, and ideal settings of nVNS, as well as how nVNS influences stroke.

### Non-invasive Vagus Nerve Stimulation Combined With Rehabilitation Improves Upper Limb Function After Chronic Stroke

It is generally accepted that upper extremity impairment as one of the results of stroke has a deep impact on quality of life, but the clinical application of the treatment may not readily be seen until after stroke. Studies have shown that iVNS paired with rehabilitation significantly improves forelimb strength and speed in models of ischemia and hemorrhage in rats (Hiraki et al., 2012; Hays et al., 2014, 2016; Khodaparast et al., 2016). Clinical studies showed that paired rehabilitation with VNS improves motor function in patients suffering from chronic strokes. The Fugl-Meyer Assessment-Upper Extremity (FMA-UE) score of stroke patients after iVNS was clearly higher than the score of pure rehab patients who did not receive iVNS (Dawson et al., 2020). Significant improvements in the Wolf Motor Function Test (both in terms of function and timing), Box and Block Test and Nine-Hole Peg Test has also been observed (Dawson et al., 2020). Similar results have also been reported in stroke patients treated with nVNS. Redgrave J.N. et al. (2018) conducted a pilot study combining taVNS with post-stroke upper limb rehabilitation in 18 sessions (1 h), showing improvement in motor function in the pilot study. While Redgrave and Baig used therapists to conduct rehabilitation training, Capone et al. (2017) have reported that taVNS combined with robot-assisted rehabilitation may be able to promote mild improvements in arm function and promote long-term benefits for stroke recovery.

Motor Activated Auricular Vagus Nerve Stimulation (MAAVNS) was devised as a closed-loop solution to the parametric problem (Cook et al., 2020). It combines taVNS with motor activity by using pulses at 25 Hz for 500 s during a focused motor task (Cook et al., 2020). It has been shown to be an effective neurorehabilitation tool and in early studies has shown promise in helping neonates learn motor skills (Badran et al., 2018, 2020). It is being explored further to facilitate stroke rehabilitation in adults. Therefore, the continued development of nVNS may radically change the field and potentially remove the barrier of surgery for many patient populations.

### Non-invasive Vagus Nerve Stimulation Improving Sensory Recovery After Chronic Stroke

Stroke survivors with sensory impairments tend to recover less functionally after their injuries. A long-term follow-up study found iVNS combined with tactile therapy improved sensory function in a man suffering from the severe sensory decline in his left hand and arm (Meyers et al., 2018). This may be related to increased neuroplasticity throughout the brain. Following the study, the authors speculated that combining VNS with sensory stimulation can be an alternative method for promoting neuroplasticity and sensory recovery for chronic stroke patients. However, this was based on only one case study. After that, Baig et al. reported the impact of taVNS paired with repetitive motor task practice on sensory recovery in a cohort of chronic stroke patients. An average of 18 sessions (1 h/session) were given over 6 weeks to twelve participants who were >3 months post-ischemic stroke and would still have residual upper limb weakness. The repetition of functional arm movements concurrently with the taVNS at the maximum level of intensity is 300 repetitions. The UFM (Upper Limb Fugl-Meyer) assessment was used to assess the light touch and proprioception of the upper limb at baseline and during post-intervention. Seven out of 11 participants (64%) who had sensory impairment at baseline regained some sensation after the intervention. Patients with the greatest improvement in motor function had the greatest increase in UFM sensation.

There is a possibility that the improvements in proprioception observed in subjects could be explained by an improvement in strength and range of motion achieved through upper limb tasks facilitated by taVNS. As a result of the increased range of joint movements, it is possible that the increased sensory feedback from the affected limb increased neuroplasticity in the cortical sensory networks. When combined with the correlation between improved motor function and sensory feedback, it is possible to hypothesize that motor and sensory recovery are positive feedback loops that mutually enhance one another.

### Non-invasive Vagus Nerve Stimulation Treating Post-stroke Insomnia After Chronic Stroke

Patients with cerebrovascular disease are often affected by post-stroke insomnia (PSI). Approximately 37–59% of patients with stroke complain about insomnia (Duss et al., 2018). Studies suggest that insomnia is also associated with an increased risk of morbidity from cardiocerebrovascular disease as well as a reduced outcome from stroke. It has been proved that taVNS is effective in treating depression with insomnia and primary insomnia (Liu et al., 2020). A case report by Zhao B. et al. (2019) examined the effectiveness and neuromechanics of taVNS in PSI patients. BOLD-fMRI was carried out before and after 4 weeks of taVNS. A 4-week taVNS intensive treatment produced significant improvement in insomnia symptoms. Falling asleep time was reduced to less than 30 min, and sleep duration was increased to 7 h. The therapeutic effect was still observed 3 months after treatment. PSQI scores dropped from 13 to 8 points.

Based on the association of the basal ganglia with the frontal lobe and thalamus, a reduced functional connectivity in the striatum and thalamus may suggest an emotional circuit disorder. Following taVNS treatment, posterior cingulate cortex and regions of basal ganglia associated with emotion showed increased functional connectivity. This case study provides evidence that taVNS therapy may provide a new, portable, self-managed, and safe technique for the treatment of PSI patients.

### Clinical Trials to Assess Safety and Efficacy of Non-invasive Vagus Nerve Stimulation After Subacute Ischemic Stroke

Researchers recently published a randomized pilot study exploring the safety and effectiveness of taVNS in treating patients with subacute ischemic stroke. In this study, 21 patients with strokes in the acute or subacute phase (between 0.5 and 3 months post onset) were included (Wu et al., 2020). At the endpoint, there were significantly greater improvements in FMA-U, FIM, and WMFT scores in the taVNS group compared with the sham-taVNS group. Moreover, the taVNS group obtained a significantly higher improvement of FMA-U score as compared with the sham-taVNS group at 4and 12 weeks. Only one adverse event related to contact with the auricular skin electrodes was noted. In the present study, taVNS proved to have a beneficial effect on the rehabilitation of upper limb motor function in patients with subacute strokes. nVNS may be able to reduce ischemic brain injury as it can be easily applied within a non-hospital setting early after stroke thanks to its relatively small therapeutic window.

### Side Effects of Non-invasive Vagus Nerve Stimulation

It has been shown that the nVNS was safe and well-tolerated, and those adverse events were very rare (Capone et al., 2017; Redgrave J.N. et al., 2018; Baig et al., 2019; Zhao B. et al., 2019; Wu et al., 2020). Redgrave J. et al. (2018) published a systematic review of the safety and tolerability of taVNS. Itching and redness (16.7%) around the stimulation site are common side effects, as are tingling and pain in the area (Redgrave J. et al., 2018). Some less common side effects have been noted in <1% of the study participants, including nausea and vomiting (Schulz-Stübner and Kehl, 2011; Kreuzer et al., 2014; Jacobs et al., 2015; Yuan and Silberstein, 2016), headache (Stefan et al., 2012; Kreuzer et al., 2014; Gaul et al., 2016; Yuan and Silberstein, 2016; Baig et al., 2019), facial drooping (Goadsby et al., 2014; Yuan and Silberstein, 2016), dizziness (Jacobs et al., 2015; Gaul et al., 2016; Liu et al., 2018; Baig et al., 2019), vocal hoarseness (Stefan et al., 2012; Goadsby et al., 2014).

In addition, due to the vagus nerve’s influence on cardiac activity, researchers closely monitored HR and BP during nVNS sessions in to detect any potential cardiovascular harm. The HR and systolic blood pressure (SBP) do not show significant pre-post differences. All cardiovascular parameters did not change significantly throughout the treatment. Heart palpitations were reported in one research (Bauer et al., 2016). According to the systematic review by Redgrave J. et al. (2018), 7/1322 participants in total reported cardiac side effects such as palpitations, arrhythmia, bradycardia, and hypotension. Steyn et al. (2013) found that the mean heart rate in four participants with asthma decreased from 106 to 85 beats per minute following nVNS. However, all participants experienced no symptoms following the procedure. Symptomatic bradycardia was observed in a male volunteer who collapsed with bradycardia and hypotension after receiving bilateral conchal taVNS (2–100 Hz, pulse width 0.2 ms) in addition to a painful stimulus (Laqua et al., 2014). Kreuzer et al. (2012) reported two cases of cardiac arrhythmia (left bundle branch block and sinus arrhythmia), in their retrospective assessment of the cardiac safety of taVNS. No work has yet examined the relationship between stimulation parameters or dose and the rate of side effects experienced, which should be a priority of future research in the area, and clear documentation of both side effects and stimulation parameters is crucial to observe any trends.

### Stimulation Parameters

For VNS, setting the optimal stimulation parameters has a huge impact on clinical efficacy. Morrison et al. (2021) found that stimulation intensity affects motor cortex plasticity. Many factors, such as the stimulation site and side, electrode and waveform configuration, continuous stimulation or pulse-synchronous stimulation, titration protocols, current amplitude and frequency, and stimulation on-and-off time can impact the clinical efficacy of VNS (De Ferrari and Schwartz, 2011). According to Helmstaedter et al. (2001), the effectors of stimulation parameters and the resulting direction of VNS’s cognitive effects appear to be highly constrained by stimulation parameters. The timing and amount of VNS therapy also play a crucial role in maximizing its therapeutic benefits (Meyers et al., 2018; Nuntaphum et al., 2018).

Due to the fact that studies have been done with participants with different clinical conditions and with diverse stimulation parameters, it is hard to determine an ideal stimulation site for any specific disease (Goadsby et al., 2014; Gaul et al., 2016; Liu et al., 2018; Martelletti et al., 2018). Despite the lack of consensus on ideal parameters, nVNS researchers carried out human clinical trials using parameters similar to those administered in cervically implanted VNS analogs.

Here are the specific parameters of stimulation for nVNS in several studies (Table 3). Most studies used the left auricular branch of the vagus nerve as the stimulated sites, except one case report chose bilateral auricular branches of the vagus nerve to stimulate. According to researchers, since vagal fibers to the heart are supposed to originate from the right side, only the left ear was stimulated to reduce the risk of cardiac side effects. A common frequency of 20 or 25 Hz is used in these studies. It is common for the stimulation current to be set according to a subject’s sensitivity or just below their pain threshold (Frangos et al., 2015; Lerman et al., 2016; Yakunina et al., 2018; Sclocco et al., 2020; Yakunina and Nam, 2021). Studies gradually raised stimulation intensity by 0.1 mA increments until the maximum level reported by participants (Redgrave J.N. et al., 2018). The intensity of stimulation ranged between 0.5 and 6 mA. Another study adjusted stimulation intensity to levels above detection thresholds and below pain thresholds (Capone et al., 2017). The range is similar to those reported in other diseases, Stimulation amplitudes vary over a wide range [from 0.5 mA (Jongkees et al., 2018) to 12 mA (Trevizol et al., 2016)]. The amplitude or amount, of energy delivered to tissues, is also unknown despite current values for electric motors being reported, due to the significant effect of electrode and tissue impedance and the need for precise placement. In addition, the electrochemistry of the stimulation electrode has a significant impact on the maximum current tolerance of the participant, without a doubt.

**TABLE 3:** Stimulation location, parameters, therapeutic effects, and side effects for all studies assessing the efficacy of nVNS in patients with stroke.

Authors	Study groups	Stimulation sites and device	Phase of stroke	Paired	Parameter settings	nVNS duration	Therapeutic effects	Side effects
Wu et al., 2020	taVNS/sham group; Randomized pilot study	taVNS; left ear concha; bhd-1a transcutaneous electrical stimulation therapy instrument (Bohua, china).	Subacute ischemic stroke	taVNS paired with conventional rehabilitation training	20 Hz; 0.3 ms; lasting 30 s each time, stimulating once every 5 min; Mean stimulation intensity 1.66 ma	15 days.	Improves upper limb motor function	Skin redness
Redgrave J.N. et al., 2018	Feasibility study with no control group.	TaVNS; left ear concha; Nemos (cerbomed)	Chronic stroke. 3 months post-stroke	taVNS paired with upper limb repetitive task-specific practice	25 Hz; 0.1 ms; Median stimulation intensity 1.4 mA	3 times a week, over 6 weeks	Improves upper limb motor function	Light-headedness in one Participant and general tiredness and fatigue in two
Baig et al., 2019	Feasibility study with no control group.	TaVNS; left ear concha; Nemos (cerbomed)	Chronic stroke. 3 months post-stroke	taVNS paired with repetitive upper limb task training	25 Hz;0.1 ms; Median stimulation intensity 1.4 mA	3 times a week, over 6 weeks	Promotes motor and sensory rehabilitation	None reported
Capone et al., 2017	Real or sham tVNS associated with Robot-assisted therapy.	taVNS; left ear concha; Twister-ebm	Chronic stroke, ischemic or hemorrhagic	taVNS paired with robot-assisted therapy	20 Hz;0.3 ms ,lasting 30 s each time, stimulating once every 5 min Mean stimulation intensity 2.0–4.5 mA	10 working days.	Improves upper limb function	None reported
Zhao B. et al., 2019	Case report	taVNS; bilateral auricular concha areas; device not mentioned	7 months post-hemorrhagic stroke	None	20 Hz; less than 1 ms; Intensity 4–6 mA	30 min, twice a day, 4 weeks	Alleviates post-stroke insomnia	None reported

## Conclusion and Future Direction

In this review, we reviewed current animal and clinical researches on non-invasive vagus nerve stimulation on cerebral stroke, emphasizing the outcomes, underlying mechanisms, stimulation parameters, sites of stimulation, and side effects.

The development of neuroscience has led to a new type of intervention, neuromodulation therapy, that targets the nervous system to achieve therapeutic results. Several studies have shown that nVNS affects the same brain regions and yields therapeutic effects similar to iVNS (Terré and Mearin, 2009; Van Leusden et al., 2015). Since nVNS is non-invasive, it has been receiving special attention in basic, clinical, and translational research for its benefits which are comparable to those of iVNS, ease of use, and reduced side effects, In addition, it is more accessible. Auricular and cervical branches of the vagus nerve are most commonly targeted by nVNS.

As nVNS continues to emerge as a promising treatment in stroke, there is still a lot to be done and a large number of literatures to be improved. Several studies have confirmed the effect of nVNS on stroke rehabilitation, however, most of the current studies focused on upper limb function, and future studies need to focus on the improvement of other functions post-stroke, such as cognition impairment, dysphagia, aphasia, and intestinal dysfunction. There is a lack of large sample RCT studies, and therefore, no strong evidence on the role of nVNS in stroke rehabilitation.

Rehabilitation effects are being demonstrated in stroke. The parameters and protocols of most of the described methods vary enormously, so there is no clear evidence on the best location to apply nVNS or the stimulation parameters that will provide the most therapeutic benefit. As nVNS research grows, we need to take a historical perspective into account and further optimize the parameter space. In addition, study results should also be analyzed to determine the frequency of treatments, the number of doses per day, and the degree of treatment tolerance.

The precise mechanism by which nVNS exerts its therapeutic effects is still unclear. We need further studies examining the mechanical basis of nVNS to facilitate our future trials. A systematic study must be conducted to reveal the precise mechanism of action and ideal stimulation modalities of nVNS if it is to reach its full potential as a non-invasive and clinically relevant therapy. Future investigations should not be restricted by past hypotheses about the effects of nVNS on neural activation and function.

Most studies have only a small sample, some with only one participant. This makes it difficult to determine whether the findings or proposed pathways can be generalized. In order to avoid the risk of having extreme or biased results, studies with a large sample size are necessary. Further standard stimulation methods of nVNS combine electrophysiology and imaging evaluation methods are needed to reduce subjective bias during training and devise more effective rehabilitation strategies for stroke.

In addition to helping avoid costly missed opportunities for reducing ischemic brain injury, nVNS may be able to reduce ischemic brain injury as it can be easily applied within a non-hospital setting early after stroke thanks to its relatively small therapeutic window.

## Author Contributions

QW conceived and supervised the project. LL and DW researched literature and wrote the manuscript. HP, LH, XS, and CH contributed to the manuscript revision. All authors contributed to the article and approved the submitted version.

## Conflict of Interest

The authors declare that the research was conducted in the absence of any commercial or financial relationships that could be construed as a potential conflict of interest.

## Publisher’s Note

All claims expressed in this article are solely those of the authors and do not necessarily represent those of their affiliated organizations, or those of the publisher, the editors and the reviewers. Any product that may be evaluated in this article, or claim that may be made by its manufacturer, is not guaranteed or endorsed by the publisher.
